# Removal of COVID-19 Spike Protein, Whole Virus, Exosomes, and Exosomal MicroRNAs by the Hemopurifier® Lectin-Affinity Cartridge in Critically Ill Patients With COVID-19 Infection

**DOI:** 10.3389/fmed.2021.744141

**Published:** 2021-10-08

**Authors:** Dennis E. Amundson, Usman S. Shah, Rosalia de Necochea-Campion, Michael Jacobs, Steven P. LaRosa, Charles J. Fisher

**Affiliations:** ^1^Department of Critical Care, Scripps Mercy Hospital Chula Vista, Chula Vista, CA, United States; ^2^Department of Critical Care, Hoag Hospital Newport Beach, Newport Beach, CA, United States; ^3^Aethlon Medical Inc., San Diego, CA, United States

**Keywords:** SARS-CoV-2, extracorporeal therapy, hemopurifier, lectin, Galanthus nivalis agglutinin, exosome, microRNA, COVID-19

## Abstract

Coronavirus−19 (COVID-19) has rapidly spread throughout the world resulting in a significant amount of morbidity and mortality. Despite advances in therapy, social distancing, masks, and vaccination many places in the world continue to see an increase in the number of cases and deaths. Viremia is commonly present in severely ill patients with COVID-19 infections and is associated with organ dysfunction and poor outcomes. Exosomes released by activated cells have been implicated in the pathogenesis of COVID-19 infection. We report the experience of two cases of critically ill COVID-19 patients treated with the Hemopurifier; a lectin affinity cartridge designed to remove mannosylated viruses and exosomes. Both patients tolerated the Hemopurifier sessions without adverse effects. In the first patient removal of exosomes and exosomal microRNAs was associated with improved coagulopathy, oxygenation, and clinical recovery, while in a second patient removal of COVID-19 by the Hemopurifier cartridge was observed. The Hemopurifier is currently under further investigation in up to 40-patients in a safety and feasibility study in ICU patients with COVID-19 infection.

## Introduction

On November 17, 2019, the first case of COVID-19, a severe respiratory infection caused by the SARS-CoV-2 virus, was described in Wuhan, China. The virus rapidly spread across the globe with ~167,391,920 million cases and 3.47 million deaths as of May 25, 2021 (https://coronavirus.jhu.edu/map.html). Fortunately, with mitigation measures, treatments and vaccinations, cases and deaths have decreased in many locales, yet many disease hotspots remain. A need to explore additional treatments, particularly in those most desperately ill, remains.

Early in the pandemic the magnitude of viremia and its clinical importance was underappreciated. A recent meta-analysis has revealed the presence of viremia in 34% of COVID-19 infected patients with higher rates occurring in the critically ill. The presence of viremia was associated with disease severity, and the development of multi-organ failure (1). COVID-19 itself is a prothrombotic virus which activates thrombus formation *via* endothelial damage and leads to the development of a COVID-19 associated coagulopathy (CAC) (2) Exosomes, extracellular vesicles ranging in size from 50 to 150 nm are released by activated cells in response to stimuli. Exosomes are involved in cell to cell signaling *via* cargo including proteins, receptors cytokines, and genetic material such as non-coding microRNAs (3). Exosomes have recently been implicated in the pathophysiology of severe infection including during COVID−19 infection (4). Viral proteins have been found in exosome cargo suggesting that COVID-19 may utilize endocytosis in cell to cell spread. MicroRNAs are involved in mRNA degradation and inhibition of protein translation (5). Differential upregulation of microRNAs during COVID-19 infection is associated with coagulation and inflammation (6, 7).

**Figure 1:**
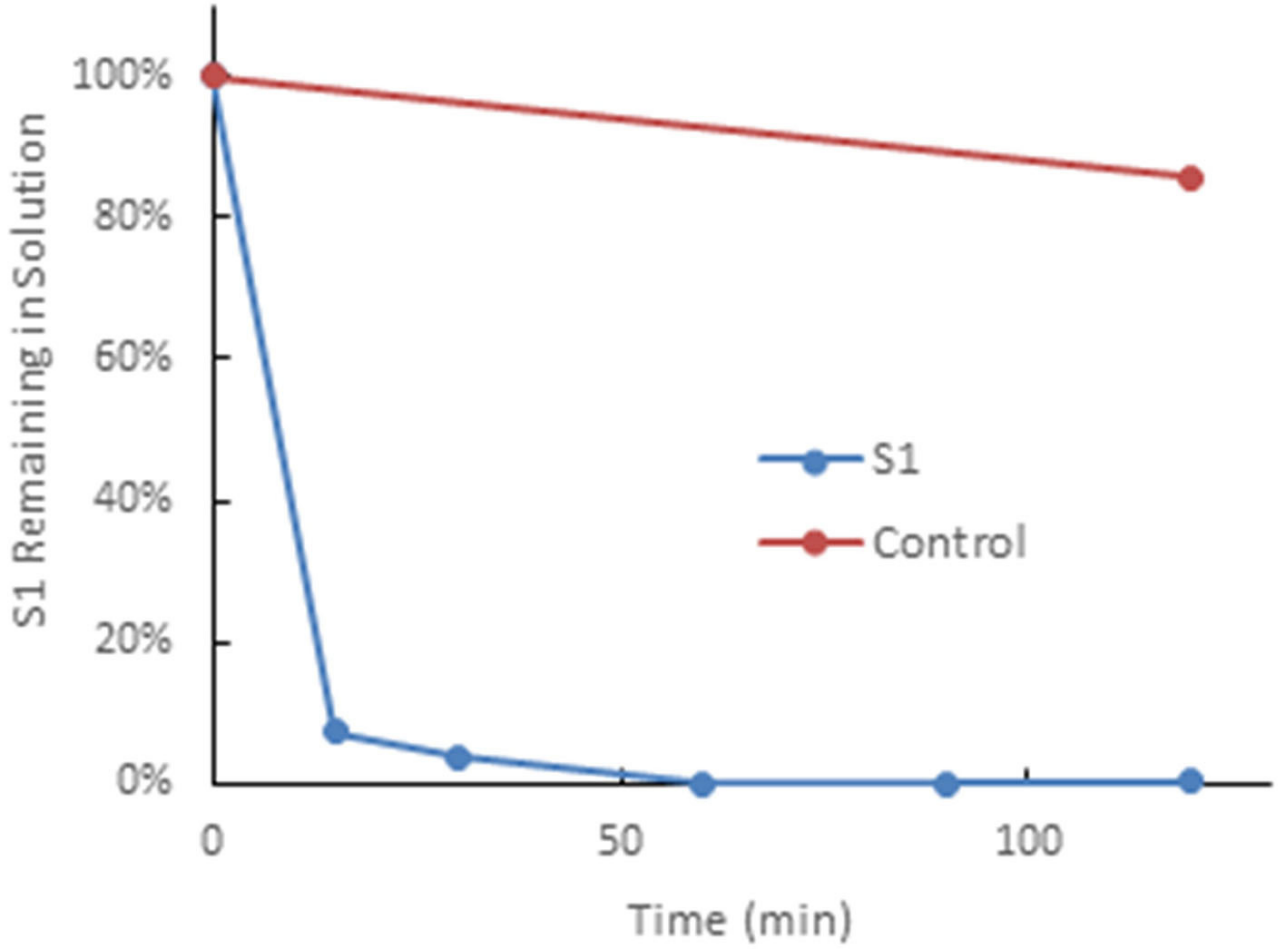
The Hemopurifier captures SARS-CoV-2 spike glycoproteins. This experiment was performed by circulating a solution spiked with the S1glycoprotein of SAR5-COV-2 over the mini-Hemopurifier column *in vitro*. Briefly, 10 mlof a 1 μg/ml solution of SAR5-COV-2 S1 in phosphate buffered saline was circulated over a Hemopurifer column containing 0.7 g of affinity resin at a flow rate of 50 ml/min. The rate of viral S1 capture, expressed as a percentage of S1 remaining in solution vs. time, was established by removing fluid samples at defined time intervals. The control consisted of S1 kept on the benchtop for the duration of the experiment (i.e., not run through the device).

The Hemopurifier® (HP) cartridge (Aethlon Medical, Inc., San Diego, CA) is a hollow fiber plasma separator filled with an affinity resin containing the lectin Galanthus nivalis agglutinin (GNA) from the Galanthus nivalis plant (the common snowdrop) (8). GNA has a high affinity to mannose-rich glycoproteins which are present on enveloped viruses as well as exosomes (9, 10). A recent *in-vitro* study demonstrated near complete the removal of the SARSCoV2 spike protein by a mini-Hemopurifier within 30 min (Figure 1).

Here, we describe the emergency use of the Hemopurifier in two cases of critically ill patients with COVID-19 infection. The report contains details of the treatments, clinical course, as wells as changes in laboratory values, plasma COVID-19 viral load and total exosome and exosomal miRNA concentrations following treatment. This report is novel in that is the first attempt to remove both virus as well as exosomes from patients with COVID-19 infection.

## Materials and Methods

### Hemopurifier Lectin-Affinity Treatment

**Figure 2:**
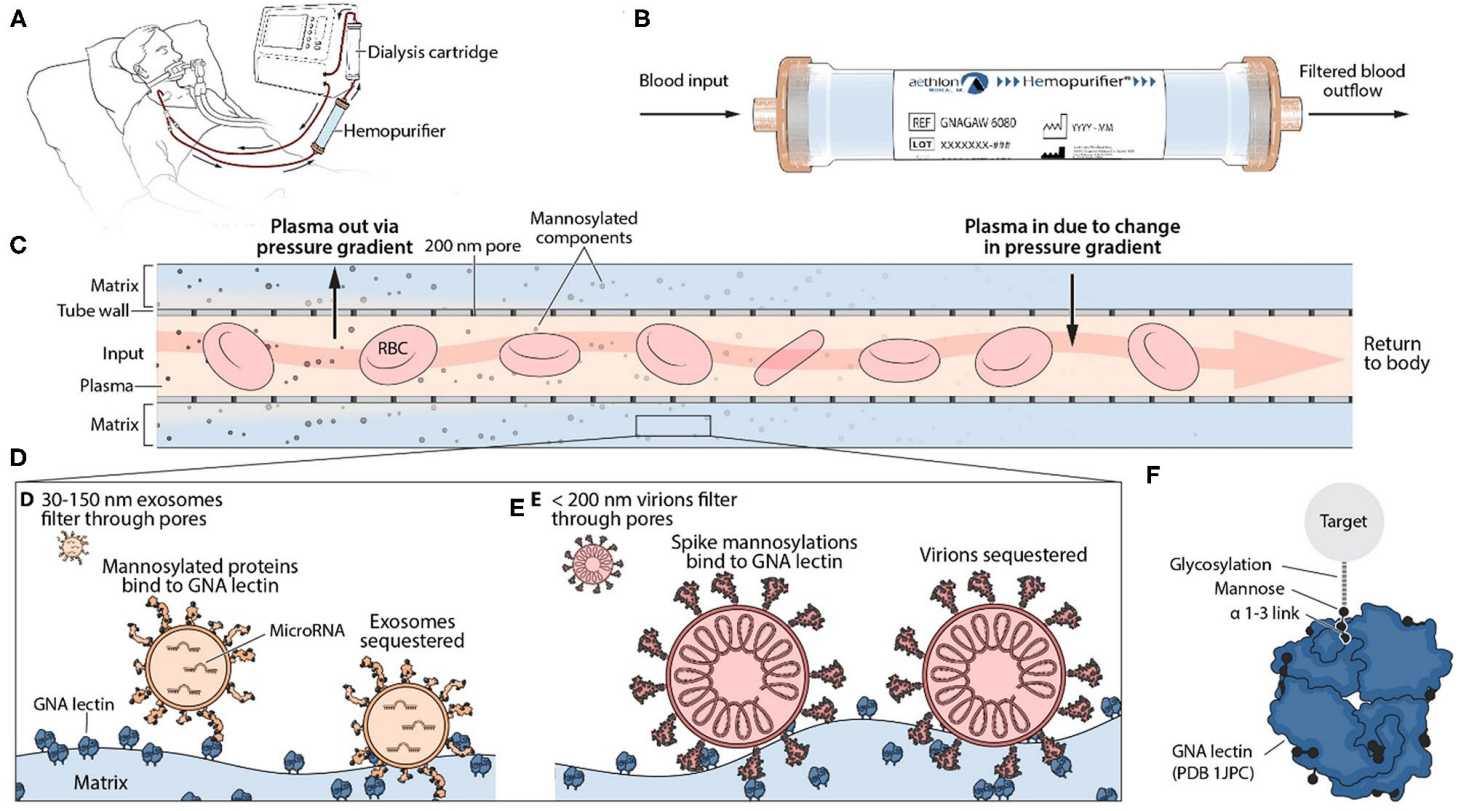
Mechanism of Action of Hemopurifier Lectin-Affinity Cartridge. **(A)** Set-up of Hemopurifier. **(B)** Blood flow through Hemopurifier. **(C)** Plasma separation and size exclusion within the Hemopurifier. **(D)** Binding of mannosylated exosomes to the GNA lectin. **(E)** Binding of mannosylated virions to GNA lectin. **(F)** GNA lectin binding to high mannose glycans with alpha 1-3 linkage.

Two patients were treated with the Hemopurifier for 6 h daily. The cartridge was operated *via* a standard dual lumen veno-venous hemodialysis catheter. Blood entered the cartridge where plasma was forced through the pores (≈200 nm) of the hollow fiber membrane due to the pressure gradient established. Blood cellular elements remained in lumen of the hollow fibers. Plasma entered the extracapillary space where the lectin-affinity resin resides. Glycosylated molecules were bound by the lectin and prevented from re-entering the circulation. The pressure gradient was reversed along the course of the hollow fibers allowing the plasma to flow backward through the hollow fibers to recombine with the blood before re-entering the circulation (Figure 2) (11).

### Laboratory Measurements

Clinical parameters and laboratories were collected as per standard of care. Blood samples for plasma COVID viral load measurement and exosomal analysis were collected in EDTA blood collection tubes prior to and after each Hemopurifier session. Plasma was isolated, deactivated with viral lysis buffer, and shipped frozen to Aethlon Medical for further analysis. The used Hemopurifier cartridge was sealed and refrigerated and then sent back for viral elution by the following methodology. First, used Hemopurifiers were flushed with a 200 ml solution of 0.5 M alpha-methylmannoside (α-MM), a lectin binding competitor (12), to gently elute a portion of the blood components bound to the resin. Subsequently, Hemopurifier cartridges were flushed with 200 ml of TRIzol reagent (Thermo Fisher) to elute any remaining blood components, proteins, and nucleic acids still bound to the GNA lectin. A spin column capture methodology was used to isolate RNA eluted in the Trizol solution. Briefly, 1 ml of the Trizol-eluent (frozen at −80°C) was thawed and mixed with 200 μl of chloroform, vortexed for 15 s and incubated at room temperature on the benchtop for 2–3 min. The mixture was then spun at 12,000 g, 4°C, for 15 min and 600 μl of the upper aqueous phase collected and transferred to a new tube. This was mixed with 1.5 × volumes (900 μl) of 100% EtOH and then added to the QiaAMP Viral RNA extraction column (Qiagen, cat#52906). The remaining RNA isolation procedure from the column was performed as instructed by the manufacturer, and purified RNA was eluted in 45 μl of AVE Buffer. A one-step RT-qPCR methodology (13) was used for detection of the SARS-nCoV2 virus with the Taqman 2019 nCov Assay Kit (Thermofisher Scientific, Cat# A47532) targeting three unique SARS-nCoV2 genome sequences (N-protein, S-protein, and ORF1ab) and the Taqman Fast Virus 1-step master mix (Thermofisher, Cat#4444434). An estimate of the viral copy number captured on the hemopurifier was calculated by relative comparison to measurements of the positive control standard (Thermofisher Scientific, #A47533).

Plasma COVID viral load testing was accomplished using the same viral RNA detection techniques. The biological specimens, plasma samples (with EDTA anticoagulant) were collected from each pre- and post-Hemopurifier therapy session, and 140 μl from each plasma specimen was processed in buffer AVL (Qiagen) to isolate nucleic acids using the QiaAMP Viral RNA extraction kit according to the manufacturer's instructions. Purified RNA samples were collected in 30 μl of AVE Buffer and 5 μl were used in each RT-qPCR to quantify SARS-nCoV2 viral copy numbers. For comparative analysis of viral loads among distinct plasma samples, calculations were further normalized to the quantity of Ribonuclease P (RNAse P) measured in each sample, a recommended methodology to control for differences in SARS-nCoV2 sample collection and processing techniques (14).

Exosomes were purified from patient plasma using an established methodology (15). Briefly, 1 ml of patient plasma was pre-cleared through a two-step centrifugation process (2,000 g for 10 min; 10,000 g for 30 min) to remove larger plasma particles, then filtered through a 0.2 uM PES membrane, and loaded onto a 10 ml Sepharose CL-2B column. Exosomes were isolated from the rest of the plasma components through size exclusion chromatography by adding 1 ml increments of PBS to the Sepharose column until the Fraction #4 eluent, containing plasma exosomes was collected. Exosome quantification data was collected using nanoparticle tracking analysis (NTA) techniques as previously described (16), using a Nanosight LM10 instrument. In order to obtain reliable quantification measurements, plasma exosome samples had to be diluted in 0.2 μM filtered PBS to a concentration of ~10^8^-10^9^ exosomes/ml. Approximately 20–100 particles could be observed in the Nanosight field of view once exosome samples had been diluted to the appropriate concentration range. Enhanced detection of smaller exosome populations isolated from the COVID plasma was achieved using a Camera Level of 12 and a Detection Threshold of 3 measurement parameters. Three 30 s capture videos of different segments of the homogenous exosome sample were evaluated with the NTA 3.3 software to obtain particle quantification and sizing measurements.

MicroRNA was isolated from the COVID plasma exosomes using the miRNAeasy Serum/Plasma RNA extraction kit (Qiagen, cat#217184) and incorporating an exogenous miRNA spike-in control (5.6 × 10^8^ copies cel-miR-39-3p miRNA/sample) to control for variability introduced during the sample preparation process. miRNA was reverse transcribed to a cDNA template using the Taqman Advanced miRNA cDNA Synthesis Kit (Cat#A28007). Specific miRNA targets were amplified on a Quant 3 qPCR machine using specific Taqman Advanced miRNA primer/probe sets (hsa-miR-424-5p, hsa-miR-16-2-3p, Cat#A25576). Quantification of miRNA sequences was done by normalization to the exogenous spike-in cel-miR-39-3p miRNA control, using ΔΔCt methods (17). MicroRNAs associated with coagulation, inflammation and acute lung injury were measured.

## Results

### Case 1

The patient was a 59-year-old female with a past medical history notable for obesity, hypertension, hyperlipidemia, alcohol abuse, and mechanical aortic valve replacement on warfarin. She was admitted in July 2020 with COVID-19 pneumonia and admitted to the general medical ward for oxygen and other therapies. She received a course of dexamethasone and was subsequently given convalescent plasma on hospital day (HD) 8. She developed progressively worsening respiratory failure and acute respiratory distress syndrome (ARDS) despite high flow nasal cannula O_2_ followed by bilevel positive airway pressure (BIPAP) therapy. She was transferred to the Intensive Care Unit (ICU) on HD 11 for intubation. After intubation, mechanical ventilation and prone positioning, her oxygenation did not improve. She was not considered a candidate for Remdesivir because of the duration of her disease, but she did receive a course of tocilizumab on HD 12 and was also administered high dose methylprednisolone. Despite all interventions, her PaO_2_/FIO_2_ ratio continued to decline to the point that she could not maintain adequate oxygenation, while paralyzed on Rocuronium and in the prone position on 1.0 FiO_2_ and positive end-expiratory pressure (PEEP) of 14 cm H_2_O. She was also treated with epoprostenol (EPO) and initially could be supinated while on it but subsequently failed supination even on EPO. She was evaluated for possible extracorporeal membrane oxygenation (ECMO) and deemed to not be a candidate.

On hospital day 21 the attending physician and an independent physician determined that the patient had failed maximal medical treatment for COVID-19. A written request was made to Aethlon Medical, Inc. for single patient emergency use of the Aethlon Hemopurifier. In accordance with federal regulations governing emergency use, all regulatory documentation was obtained including signed informed consent as well as IRB approval. On HD 22 the patient received her 1st Hemopurifier treatment. Prior to her 1st HP treatment, she was on maximal ventilatory support (FiO_2_ 1.0, PEEP 14 cm H_2_O, proned). The venous side of the double lumen internal jugular catheter clotted 20 min into the first Hemopurifier treatment. Patency was restored with TPA. The treatment was re-started with a fresh Hemopurifier cartridge, and she successfully completed the prescribed 6-h treatment. She received a 6-h HP treatment once daily 4 days over hospital days 22–25. The patient tolerated the procedure well without evidence of allergic reaction, thrombotic complications, or hemolysis.

The clinical impression of the attending physician following the four treatments was that there was a slight improvement in her clinical status. A review of the patient's laboratories (Table 1) reveals that, prior to treatment, she had evidence of COVID-induced coagulopathy (CAC) with thrombocytopenia and a markedly elevated D-dimer level. The patient also had marked respiratory impairment as indicated by a low PaO_2_/FIO_2_ ratio, systemic inflammation as indicated by hyperferritinemia and tissue damage as indicated by an elevated lactate dehydrogenase (LDH). On HD 27, essentially 2 days after the first 4 HP treatments, the patient had improvements in her markers of coagulation and oxygenation as well as decreases in her ferritin and LDH.

**Table 1:** Laboratory values over time in emergency use case #1.

**Date**	**D-dimer (ng/ml)**	**Platelet (cells/mcl)**	**PT/INR**	**Ferritin (ng/ml)**	**Lactate (mmol/l)**	**PaO_**2**_ /FIO_**2**_ ratio**	**FI0_**2**_**	**Tidal Volume**	**PEEP**	**ALC (absolute lymphocyte count) (cells/mcl)**	**LDH (U/L)**
HD 14 (8 days prior to therapy)				3599.5 (systemic inflammation)							2370 (tissue injury)
HD16 (6 days prior to therapy)	>7,650										
HD 18 (4 days prior to therapy)		115,000			3.6 (tissue hypoxia)						
HD 22 (Day 1 therapy)			1.2 (13.6 s, prolonged)			93	0.70	400 (~6 mkl/kg)	20	780 (lymphopenia)	
HD 23 (Day 2 therapy)					2.3	98	1.0	400 (~6 mkl/kg)	20		
HD 24 (Day 3 therapy)						75.5	0.90	400 (~6 mkl/kg)	20		
HD 25 (Day 4 therapy)						88.57	0.70	400 (~6 mkl/kg)	20		
HD 27 (Day 5 therapy)	3703	162,000	1.0 (11.3 s, improved)	622.4	0.8 (normal)	136.25	0.80	400 (~6 mkl/kg)	20	1180	978 (improved)
HD 30 (8th day of therapy)						127	0.60	380	20		
HD 35 (5 days after therapy completed)						175	0.6	380	20		
HD 42 (12 days after therapy completed)						198	0.5	380	20		
HD 49 (19 days after therapy completed						152	0.5	380	10		

Given the signs of clinical improvement, as well as the thinking that exosomes may still be contributing to the patient's ongoing critical illness, the decision was made to continue the HP treatments for 4 additional days. The patient received 4 additional 6-h treatments from HD 27 through 30. The patient received a low tidal volume (~6 ml/kg) mechanical ventilation strategy for ARDS throughout her ICU stay. Following the last Hemopurifier treatment the patient's FIO_2_ was able to be weaned to 0.60. She subsequently required a tracheostomy on HD36. The patient's PaO_2_/FIO_2_ ratio continued to improve during her hospitalization. She was discharged to a long-term care on hospital day 58 with an O_2_ saturation of 98% on pressure support ventilation with an FIO_2_ of 0.45 and 5 of PEEP.

**Figure 3:**
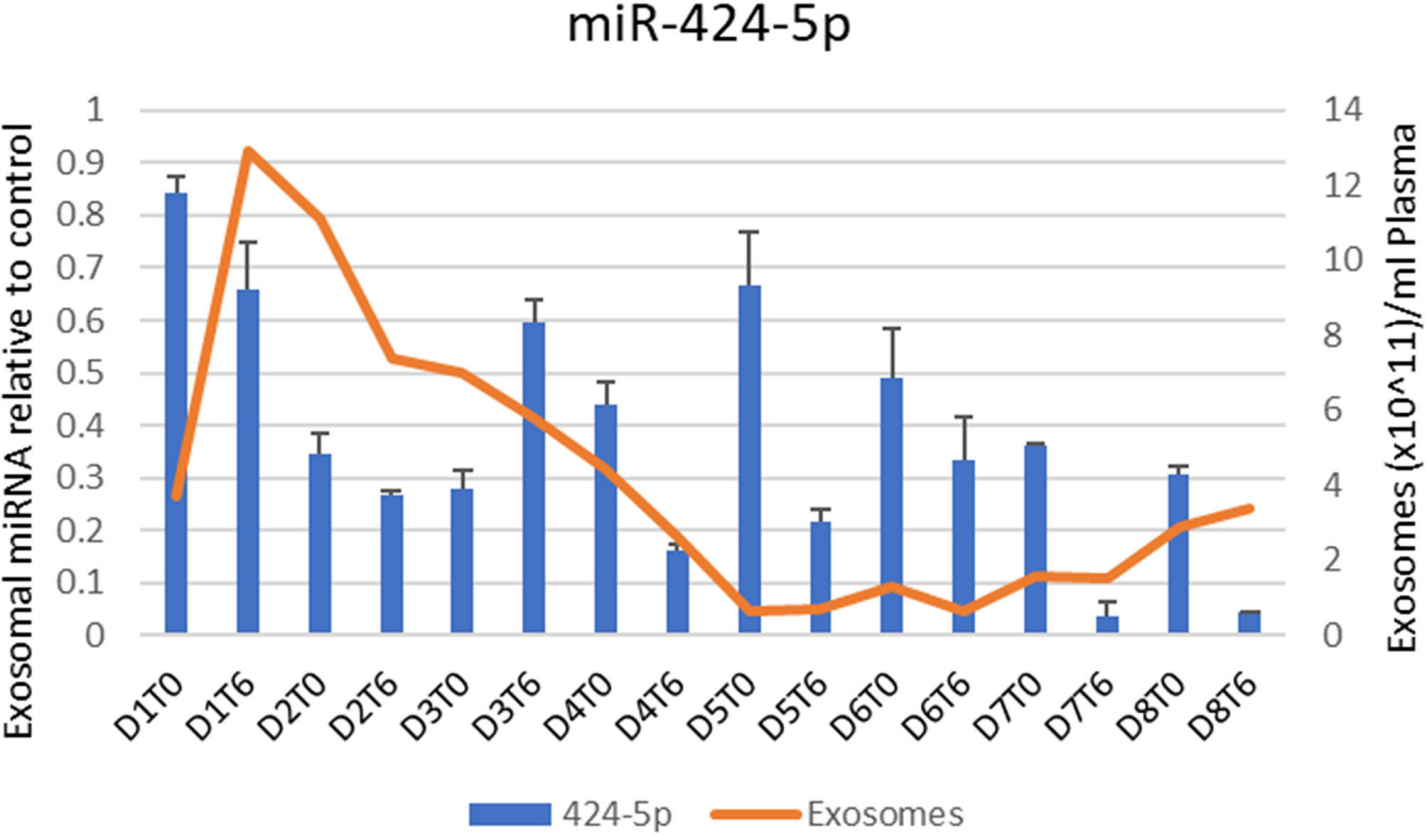
Exosome and Exosomal miR-424 quantification over time in Case 1.

**Figure 4:**
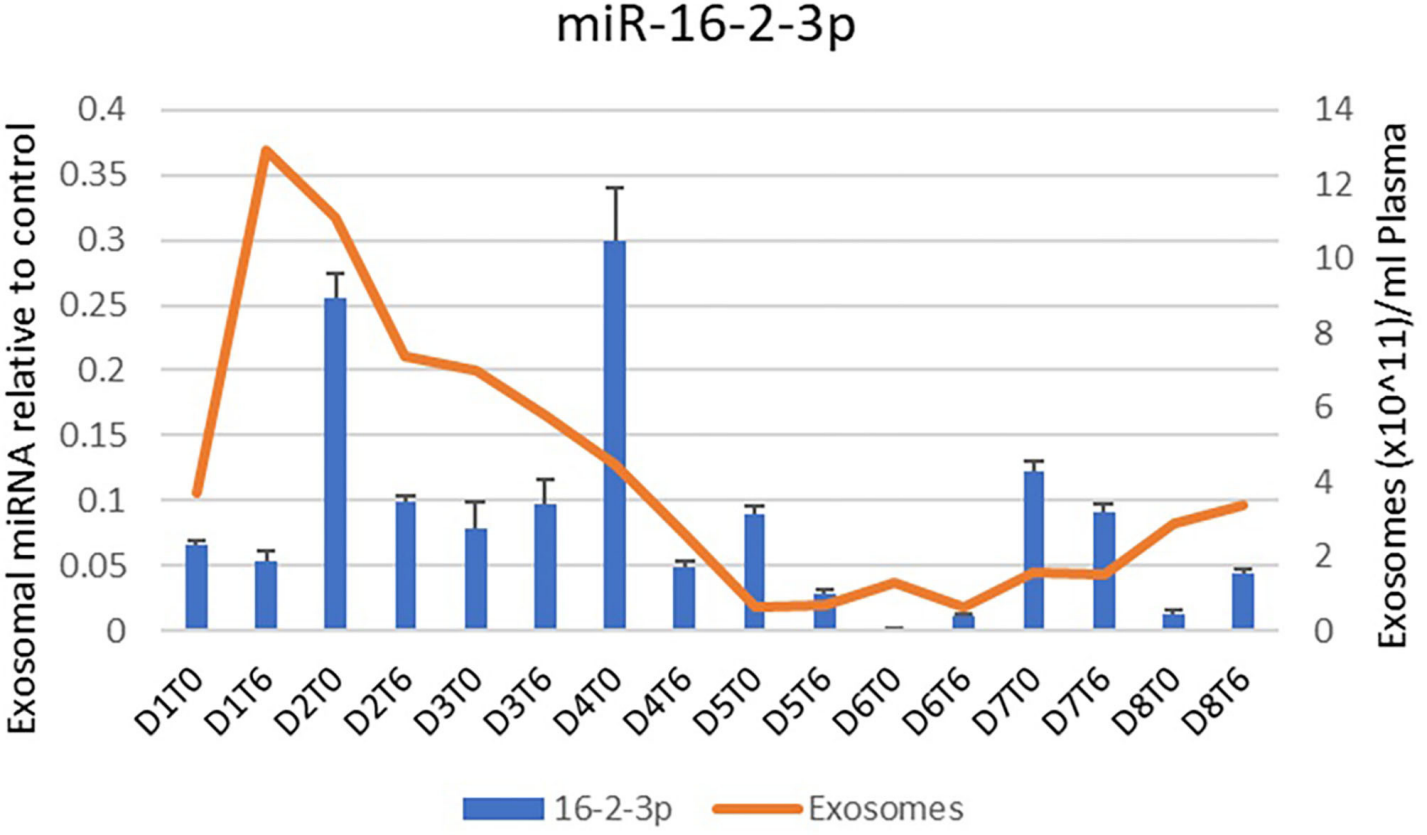
Exosome and Exosomal miR-16-3-3p quantification over time in Case 1.

Throughout the Hemopurifier treatment the patient had plasma samples stored for retrospective analysis of plasma COVID viral load as well as exosomal analysis. Blood samples were collected before and after the emergency-use Hemopurifier treatment (6 h/treatment) conducted on 8 different days. The patient's COVID-19 plasma viral load was undetectable at the onset of treatment with the Hemopurifier. Over days 2–7 of the HP treatment the total exosome concentrations decreased from pre- to post-HP treatment. Interestingly, the total exosome concentration increased during treatment on Days #1 and #8 (Figures 3, 4). A consistent pattern of decreasing exosomal miR-424 concentrations from pre- to post-HP treatment was observed over the 8 Hemopurifier treatments coinciding with the improvement in coagulopathy. The concentration of exosomal miR-16 dropped over the first 4 Hemopurifier treatments and then stayed at low levels as the patient's acute lung injury improved (Figures 3, 4).

### Case 2

The patient was a 67-year-old gentleman with a history of tetralogy of Fallot repair, coronary artery disease, and newly diagnosed diabetes mellitus. He presented to the hospital in January 2021 with a 1-week history of cough and shortness of breath. He was found to be COVID-19 positive by polymerase chain reaction test (PCR) and was admitted to the hospital. The patient was also noted to have acute kidney injury. Despite treatment with Remdesivir, Dexamethasone, Baricitinib, convalescent plasma, and full dose anticoagulation, the patient developed worsening multiple organ system failure. He was on mechanical ventilation with an FIO_2_ of 100% and 12 of PEEP, a single vasopressor for hypotension and CRRT for acute renal failure. Given the patient's deterioration despite maximal medical support, the treating physician requested the single patient emergency use of the Aethlon's Hemopurifier on HD 8. In accordance with federal regulations governing emergency use, all regulatory documentation was obtained including signed informed consent as well as IRB approval.

The Hemopurifier treatment was performed on hospital day 9. Prior to the treatment the patient required two vasopressors for hypotension as well as prone position ventilation with an FIO_2_ of 0.90 and a PEEP of 8 to maintain oxygenation. The pre-treatment SOFA score was markedly elevated at 13 indicating a predictive mortality of > 80%. The patient received 6 h and 15 min of Aethlon Hemopurifier treatment in series with CRRT. The patient had fluctuations in his oxygenation and blood pressure during the completed HP session. The patient was disconnected from the Hemopurifier without incident. An examination of the Hemopurifier cartridge did not reveal changes suggesting clotting or hemolysis and his haptoglobin level was normal.

An examination of the patient's post-Hemopurifier session labs revealed evidence of clinical worsening with a C-Reactive Protein test (CRP) that had increased from 7.9 to 16.2 mg/dl. The d-dimer level and LDH both increased to beyond the upper limit of detection. Following disconnection of the Hemopurifier the attending physicians elected to change out and reinitiate his CRRT circuit. Approximately 15 min after the new CRRT circuit was placed the patient's blood pressure began to drop. The patient developed refractory shock and refractory hypoxia and expired due to a pulseless electrical activity (PEA) arrest.

The Hemopurifier cartridge used on the patient was saved for analysis, and plasma samples for viral load testing were collected before and after the Hemopurifier session. PCR testing done on eluent from the Hemopurifier demonstrated viral capture by the cartridge. Additionally, the plasma COVID viral load normalized for RNAse P decreased by 58% in the sample collected at the end of the Hemopurifier treatment compared to the pre-treatment sample (Table 2).

**Table 2:** Hemopurifier viral capture and change in plasma viral load in case 2.

	**1 PCR R × n**	**Total RNA**	**Total trizol**		**Hemo purifier Total**
**Estimate hemopurifier capture**					
	5 ul/r × n	60/5 ul	200/1 ml		Eluted copies
HP-P2Eluent	242.1	×12	×200		5.8E + 5
	**1 R** **×** **n**	**Total RNA**	**Plasma**	**RNAsep**	**Plasma**
**Plasma viral copies normalized to RNAsep**					
	5 ul	45/5 ul	1/0.14 ml	Normalization	Copies/ml
Pre-plasma	24.3	×9	×7.14	×1	1558.6
Post-plasma	29.5	×9	×7.14	×0.34	648.1

## Discussion

In this publication we describe the first use of a Hemopurifier lectin-affinity plasmapheresis cartridge in two critically ill patients with severe COVID-19 infection. A total of 9 Hemopurifier sessions were tolerated by these two patients despite multi-organ system failure. The cases build on our experience in a critically ill patient with Ebola where viral removal and clinical improvement was observed following a Hemopurifier treatment (11). Previously the Hemopurifier had been studied in 29 ambulatory patients with > 90 Hemopurifier exposures in clinical studies involving patients with either Hepatitis C (18) or HIV. The safety profile was benign in these subjects with a side effect profile that is typical for other extra-corporeal therapies.

The first of the two reported cases is notable for the clinical course following Hemopurifier treatments observed in a patient without demonstrable COVID-19 viremia. The absence of viremia was not unexpected given that the patient received her first Hemopurifier treatment on hospital day #22. Following the initial 4 treatments the patient had evidence of improvement in COVID-19 associated coagulopathy (CAC), lung injury, inflammation, and tissue injury. We hypothesize that these improvements were due to the removal of exosomes with noxious microRNA cargo by the Hemopurifier. In our patient we observed decreases in total exosomal concentration from pre to post-Hemopurifier treatment over days 2–7. A rise in exosomes was noted on the first and eighth day of treatment. We hypothesize that the rise on the first day may have been related to the clotting in the hemodialysis catheter. The rise on total exosome concentration of the eighth day is unexplained and requires further investigation. The net rise in total exosome concentration could possibly be driven by production of exosomes carrying reparative microRNA cargo.

We specifically examined the microRNA cargo of the exosomes during the Hemopurifier treatments. A decrease in miR- 424 following the initial 4 days of HP treatment at a time when the patient's coagulopathy improved. Gambardella and colleagues found that exosomal miR-424 was upregulated in COVID patients with coagulation activation as indicated by elevated D-dimer levels vs. COVID-19 infected patients with normal D-dimer levels (6). We also noted decreases in miR-16 concentrations throughout the treatment period at a time when the patient's oxygenation was improving. In a rat LPS challenge model of acute lung injury, transfection with an miR-16 overexpression virus was associated with an increased wet/dry ratio of lung tissue and higher NFkappa B levels compared to rats transfected with an empty vector (19). These finding suggests that benefits from the Hemopurifier in COVID-19 may extend beyond viral removal and may be a result of the elimination of mannosylated exosomes.

The second case is novel in that we demonstrate for the first time the *in vivo* removal of COVID−19 from the bloodstream in an infected patient by the Hemopurifier. We were able to elute COVID-19 from the used Hemopurifier cartridge as well as demonstrate a 58% reduction in plasma COVID-19 viral load following the single 6-h treatment. Unfortunately, this patient's disease was quite advanced at the onset of the Hemopurifier therapy, and he succumbed to multi-organ failure. It is possible that viral load reduction earlier in his course of COVID-19 might have provided a clinical benefit in this patient. In a study by Bermejo-Martin and colleagues, the presence of viremia was associated with a dysregulated immune response and development of coagulopathy (20). Post-mortem studies have demonstrated viral dissemination and seeding of organs including the bone marrow, heart, and gastrointestinal tract (21). In a Swedish cohort of critically ill COVID-19 patients, the presence of viremia was associated with the need for renal replacement therapy and poor clinical outcome (22).

In summary, we describe the first two cases of critically ill COVID-19 patients treated with the Hemopurifier lectin-affinity plasmapheresis cartridge. The two patients tolerated a total of nine 6-h Hemopurifier treatments without side effects. The first patient experienced a thrombus within the dialysis catheter at the beginning of the 1st Hemopurifier treatment which was likely precipitated by COVID-19 associated coagulopathy (CAC). For the first time, we demonstrate the removal of COVID-19 from a viremic patient by the Hemopurifier. Additionally, total exosome concentrations and noxious exosomal microRNAs associated with coagulopathy and acute lung injury decreased with Hemopurifier treatments and was associated with clinical improvement in one patient.

The authors acknowledge limitations to our case series. The paper describes the treatment of only two patients with the Hemopurifier and lacks a control arm. In the 1st case where the patient improved from acute lung injury it should be acknowledged that a low tidal volume mechanical ventilation strategy was used. In a randomized clinical trial in patients with ARDS this mechanical ventilation strategy was shown to improve mortality (23). We examined only a limited number of microRNAs which could impact the pathogenesis of COVID-19 infection in our present study. As an example, we did not measure the concentration on miR-98-5p which inhibits the expression of transmembrane protease serine 2 (TMPRSS2) which has been implicated in the pathogenicity of COVID-19 (24). Important unanswered questions regarding the Hemopurifier cartridge include the optimal timing of the intervention as well as the number and duration of Hemopurifier sessions necessary to see a benefit during COVID-19 infection. An IDE safety and feasibility study enrolling up to 40 ICU patients with COVID-19 (NCT04595903) is currently underway.

## Data Availability Statement

The original contributions presented in the study are included in the article/supplementary material, further inquiries can be directed to the corresponding author.

## Ethics Statement

The studies involving human participants were reviewed and approved by the Scripps Mercy Chula Vista Hospital and Hoag Hospital Newport Beach. The patients/participants provided their written informed consent to participate in this study. Written informed consent was obtained from the individual(s) for the publication of any potentially identifiable images or data included in this article.

## Author Contributions

DA and US were the attending physicians for the patients treated, participated in evaluation of the data, and editing the manuscript. MJ and CF provided technical support on the Hemopurifier as well as evaluated the data and edited the manuscript. SL evaluated the clinical and laboratory data as well as wrote the first draft of the manuscript. RN-C performed the viral load, exosome, and exosomal microRNA assays. All authors contributed to the article and approved the submitted version.

## Funding

Aethlon Medical Inc. supplied the hemopurifiers used in this case and funded the exosomal assays and COVID-19 viral load measurements performed.

## Conflict of Interest

RN-C, MJ, SL, and CF are employees of Aethlon Medical, Inc.; the manufacturer of the Aethlon Hemopurifier. The remaining authors declare that the research was conducted in the absence of any commercial or financial relationships that could be construed as a potential conflict of interest.

## Publisher's Note

All claims expressed in this article are solely those of the authors and do not necessarily represent those of their affiliated organizations, or those of the publisher, the editors and the reviewers. Any product that may be evaluated in this article, or claim that may be made by its manufacturer, is not guaranteed or endorsed by the publisher.
